# Cultivating conscience: Moral neurohabilitation of adolescents and young adults with conduct and/or antisocial personality disorders

**DOI:** 10.1111/bioe.12849

**Published:** 2021-02-20

**Authors:** Nancy Tuck, Linda MacDonald Glenn

**Affiliations:** ^1^ Albany Medical College Alden March Bioethics Institute Albany New York United States; ^2^ Molloy College Philosophy Department Rockville Centre New York United States; ^3^ University of California Santa Cruz, Crown College Santa Cruz California United States; ^4^ California State University Monterey Bay Seaside California United States

**Keywords:** antisocial personality disorder, conduct disorder, mandatory mental health treatment, moral intuition, morality development, neurohabilitation, psychopathology treatment

## Abstract

Individuals diagnosed with conduct disorder (CD) in childhood and adolescence are at risk for increasingly maladaptive and dangerous behaviors, which unchecked, can lead to antisocial personality disorder (ASPD) in adulthood. Children with CD, especially those with the callous unemotional subgroup qualifier (“limited prosocial emotions”/DSM‐5), present with a more severe pattern of delinquency, aggression, and antisocial behavior, all markings of prodrome ASPD. Given this recognized diagnostic trajectory, with a pathological course playing out tragically at the individual, familial, and societal level, and lack of effective remediation via current standards of care, we posit an alternate treatment approach; case‐specific compulsory moral habilitation aimed at rectifying the undeveloped affective domain of adolescents and young adults suffering from these disorders. We begin with a brief historical overview of response to mental illness, review CD and ASPD diagnostic criteria and treatment limitations, and posit a unique neurohabilitative approach that focuses on the absent affective moral development of these populations. Next, we invoke a public health safety argument to justify case‐specific compulsory moral habilitation, discuss neurotechnologies to be considered in treatment, and conclude with ethical considerations and suggestions for further research.

## I INTRODUCTION

The interest in this topic is motivated by the authors’ respective professional experiences working with emotionally challenged adolescents and young adults. The first author’s extensive clinical experience counseling adolescents in hospitals and school settings has highlighted the distinctions in behavioral profiles and treatment implications between those demonstrating remorse following episodes of impulsive, explosive behaviors, and those devoid of any culpability, boasting satisfaction at having perpetrated calculated acts of harm. The second author’s experiences as a trial attorney and *guardian ad litem* for juveniles in the justice system prompted her to do her own research and connect with the first author.

The mental health and legal systems end up dealing with these individuals in a punitive manner, after harms have already been committed. But the authors assert that, in light of recognizable behavioral trajectories, a preventative treatment approach should, and finally can, be the first recourse.

## II HISTORICAL PERSPECTIVE

The history of treatment for mental illness has been plagued by centuries of superstition, ignorance, and inhumanity. People exhibiting bizarre or deviant behaviors were often viewed as spiritually “sick”, “guilty”, or “possessed by the devil”, requiring religious or punitive interventions. In the 18th century, medical generalists with peripheral interests in mental disorders, colloquially referred to as “mad‐doctors”,^1^ would make home visits to consult on family members exhibiting strange behaviors; family members who were kept out of public sight. But, with the dawn of the industrial revolution and people flocking to urban centers for work, mental illness became a more visible, and hence “societal” problem, as individuals exhibiting strange or frightening behaviors ended up in prisons or on the streets.

The next societal response to the chronically and acutely mentally ill would take the form of institutionalization in state‐run mental facilities that became “dumping grounds” for populations of all ages and manner of physical, mental, and/or developmental disability. Response to mental illness would shift from “management” to “treatment” in the late 18th, early 19th centuries, with the introduction of psychoanalysis and talk‐therapies, and again in the 1950s, through pharmacologic administration of chlorpromazine (Thorazine) for the treatment of psychoses (e.g., in schizophrenia). But news reports of electroconvulsive shock therapies and psychosurgeries (lobotomies) performed with questionable consent practices in hospitals like Willowbrook^2^ and Pilgrim State, sparked public outrage and cries for reform, leading to a protracted period of deinstitutionalization, and an overhaul of mental health treatment provision.

People living in societies are inculcated by the norms of societies in which they live—from the way they are raised, to the interpersonal behavioral responses expected. If behavioral norms are solely societal constructs, then, is it “just” that an individual who does not meet with a community’s behavioral standards should be deemed mentally ill, be medicated, or even locked up? Though this certainly presents an argument in favor of personal liberty, an equally strong rebuttal in favor of societal “contract” posits that, as members of a community, some behavioral deviance from the proscribed norm is acceptable; however, once the deviant behaviors present potential or actual harm to self or others in the community, legally sanctioned protections (e.g., involuntary psychiatric hold, sometimes called a “civil commitment”) may be invoked.

## III DEVELOPMENT OF MORALITY

Developmental psychologist Lawrence Kohlberg posited that moral deliberative ability does not fully emerge until late adolescence.^3^ But studies of infants and toddlers bear out that early moral intuitions seem to manifest long before human capacity for complex reasoning is neurologically developed.^4^ Studies show that infants are sensitive to the valence of third‐party social interactions, displaying a preference for prosocial agents over antisocial or neutral ones.^5^ This was demonstrated when infants observed puppets either helping or hindering other puppets; infants as young as 3‐month‐olds gazed significantly longer at the helpful puppets.^6^ Likewise, 6‐ and 10‐month‐old infants exhibited longer gaze time, and actively reached towards prosocial characters, not the antisocial ones. These “third‐party moral evaluations are thought to rely on intuitive processes that constitute the foundations for an innate moral core shaped by natural selection to facilitate social affiliation and collaboration” (p. 156).^7^

3–4‐year‐old children consistently judge antisocial transgressions (e.g., hitting another child) not only as wrong, but wrong across situational contexts.^8^ Children observing other conspecifics being harmed triggers empathic concern, “particularly when the harm is inflicted intentionally and is unjustifiable. Empathic concern is critical in moral cognition” (p. 158).^9^

Neuroscientific studies employing functional magnetic resonance imaging (fMRI), eye tracking, and pupillometry of children and adolescents^10^ support a “gradual maturation and integration across distinct neural computations in service of moral evaluation” (p. 159).^11^ When functional MRI studies are conducted with children and adolescents manifesting disruptive psychopathic traits, it has been demonstrated that these individuals, when viewing the suffering of others, exhibit reduced activity in brain regions typically implicated in affective responses to others’ pain; these regions include the anterior cingulate cortex, insula, and amygdala.^12^

## IV DIAGNOSTIC CRITERIA

### IV.1 A. Conduct disorder

Conduct disorder (CD) in children is defined as a repetitive and persistent pattern of behavior that violates the rights of others and age‐appropriate societal rules.^13^ According to the Fifth Edition of the *Diagnostic and statistical manual of mental disorders* (2013),^14^ a diagnosis of CD requires the presence of three of 15 criteria (behaviors) manifesting within the previous 12 months, at least one of which has presented for the past 6 months. These 15 behaviors are categorized into four dimensions: aggression to people and animals; destruction of property; deceitfulness or theft; and serious violation of rules (e.g., running away from home). For a diagnosis of CD, the behavioral symptoms must cause clinically significant impairment in social, academic, and/or occupational functioning. An additional specifier of “limited prosocial emotions” was introduced to the DSM‐5 to describe a subgroup of those with CD who also present with callous unemotional (CU) traits. To qualify for this specifier, a child must have displayed at least two out of four characteristics persistently over the previous 12 months across multiple relationships and settings. These characteristics are: lack of remorse or guilt, callousness (e.g., lack of empathy), lack of concern about performance (e.g., in school), and shallow or deficient affect. Individuals who meet criteria for the limited prosocial emotions specifier are more likely to engage in aggression that is planned for instrumental gain.^15^

It is important to note that within the CU subgroup specifier, it is the affective, or emotional, component of empathy that is absent or impaired, not the cognitive or reasoning ability (e.g., perspective‐taking, assessing others’ thoughts, theory‐of‐mind or mentalizing). This functional impairment has been associated with reduced amygdala and ventromedial prefrontal cortex responsiveness to distress cues.^16^ Developmental accounts of CU traits place primary emphasis on biologically based factors;^17^ CU traits are associated with functional abnormalities in brain regions involved in the processing of basic emotional salience, reinforcement learning, and emotion regulation (e.g., amygdala, ventromedial, prefrontal, orbitofrontal cortex, and caudate^18^). Disorders often comorbid with CD include ADHD, anxiety, depression, and substance abuse.^19^

Adolescence is a dynamic, developmentally important period of life, characterized by sweeping changes of physical maturation, increased reliance on social/peer interactions, and significant physiologic brain development. These social‐relational and neurodevelopmental processes significantly impact adaptive functioning during adolescence and emerging adulthood.^20^ Prosocial, and associated negative emotions of guilt and shame, are thought to be of particular importance for the maturing adolescent, serving to maintain attachments, and acting as “‘social regulators’ that encourage a balance between one’s self‐interested motivations and the rights and needs of others” (p. 51).^21^ Here, a distinction between guilt and shame must be made; guilt is associated with self‐blame related to one’s own behavior, whereas shame is associated with self‐blame and a pervasive view of one’s global self as “faulty”.^22^ This distinction is important, due to the different ensuing responses and behaviors. Guilt is associated with feelings of regret and remorse and is the counterpart to prosocial tendencies associated with empathy. Conversely, shame is thought to be associated with feelings of helplessness, and a desire to hide or escape.^23^

### IV.2 B. Antisocial personality disorder

A diagnosis of CD in childhood or adolescence often precedes that of ASPD in adulthood.^24^ While not all cases of CD progress to a diagnosis of ASPD, “empirical studies have demonstrated a strong link between the two” (p. 19).^25^ Antisocial personality disorder (ASPD) describes individuals with a pervasive pattern of disregard for, and violation of, the rights of others that begins in childhood or early adolescence and continues into adulthood. Epidemiological studies report a prevalence of 2–3% in the general population, with estimates of approximately 3% in men and 1% in women. In prison samples, studies have found rates of ASPD to be 47% in men and 21% in women.^26^

Often the terms sociopath, psychopath, and ASPD are used interchangeably, but they are not the same; this confusion likely stems from overlapping traits. Antisocial personality disorder has “perhaps the most overlap with the construct of psychopathy” (p. 427).^27^ Psychopathy, while not recognized in the diagnostic criteria of DSM‐5, describes individuals with many of the features of ASPD, but who manifest additional characteristics such as superficial charm, manipulativeness, callousness, and shallow affect.^28^ Evidence from a growing body of research suggests that there are variants of psychopathy; variants that are phenotypically similar, but “primary psychopathy is underpinned by a (heritable) affective deficit, whereas secondary psychopathy reflects an (environmentally acquired) affective disturbance” (p. 395).^29^ Since the secondary psychopath’s callous behavior can be understood as an emotional adaptation to childhood trauma (e.g., caretaker rejection or abuse), secondary psychopaths are viewed as more amenable to treatment than primary psychopaths.^30^

For years, attempts have been made to treat antisocial individuals using a variety of clinical approaches, including individual and/or group therapies, behavior modification programs, parent/family education, and medication administration. Unfortunately, though, there are still no truly effective treatments available for these populations. Many clinicians have adopted the position that antisocial individuals, especially those with elevated levels of psychopathy, are “so difficult to treat as to be next to untreatable” (p. 264).^31^

## V PUBLIC HARM AVERSION

Western society’s emphasis on individual liberty aligns with a medical ethical emphasis on patient autonomy. When a patient is deemed to have decision‐making capacity, she can refuse medically beneficent care for herself, although she cannot exercise the same for her minor child, wherein courts will intervene on behalf of a minor who is at‐risk of harm. Adults diagnosed with psychiatric illness retain decision‐making autonomy, unless they exhibit threat of imminent harm to self or others, at which point they can be involuntarily hospitalized. There is also a professional “duty to warn” anyone being threatened of harm when such a threat is known.^32^

Personality disorders, with ASPD the most common category, are very prevalent in the prison populations.^33^ And offenders with personality disorders, especially of a psychopathic type, exhibit a higher risk for violent crime.^34^ In 1999, the UK government introduced the Dangerous and Severe Personality Disorder (DSPD) program “to deal with a group of individuals who … were at the boundary between the health and criminal justice systems” (p. 325).^35^ DSPD is not a clinical classification, but a determination that an individual presents a significant risk of inflicting serious physical or psychological harm upon others, a risk‐factor functionally linked to a personality disorder. In spite of opposition by many in the mental health and legal communities, the DSPD program exemplifies a health policy initiative wherein public safety concerns supersede individual autonomy in cases of those meeting specific diagnostic criteria and extremes of behavior.

Considering the high percentage of incarcerated individuals suffering from ASPD, and the lack of treatment effectiveness (i.e., high rates of recidivism) when individuals with ASPD end up in the mental health system, there is an urgency to consider alternative treatment strategies in efforts to ameliorate costs and burdens on the mental health, criminal justice, and emergency response systems. People suffering from these serious personality disorders are the least likely to seek out, or comply with, treatment strategies due to narcissistic tendencies, lack of remorse, and externalization of blame.

In light of these factors, we argue that case‐specific compulsory moral neurohabilitation would be justified, so long as treatment/safety protocols are in place. Too many atrocities have occurred via medical, governmental, and religious abuses of power in the name of societal “good” (e.g., eugenics, human experimentation, incarceration of political dissidents, etc.). Understandable concerns arise from fears of diagnostic‐creep, political influence, religious, and/or social pressures. Adequately addressing these concerns will require implementation of multiple procedural safeguards, including professional diligence, empirically verified treatment strategies, ongoing case review, and accountability. Only with these “ground‐rules” in place, do we propose that there may be “instances where a compulsory moral enhancement might actually be more responsible of the state than leaving such interventions up to individual choice” (p. 203).^36^ We agree with Wiseman who asserts that it “is not whether the intervention is voluntary or compulsory that gives it decisive moral significance, but rather it is the facts on the ground that determine whether the intervention should be voluntary or compulsory” (p. 203).^37^

## VI PROPOSED NEW TREATMENT FOR MORAL NEUROHABILITATION

### VI.1 A. Habilitation

Persson and Savulescu (2008) have posited that there is “an urgent imperative to enhance the moral character of humanity” (p. 162)^38^ due to modern‐day ability to wreak havoc on a global scale (e.g., bioterrorism), as compared to ancient hunter‐gatherer/agricultural societies with limited tools, weapons, and reach of harm. But, while we recognize that there are ever‐widening gaps between human (biological) evolutionary adaptive processes and ethical challenges posed by rapid technological advancements, we do not argue for species‐wide enhancement or intervention in this paper. Rather, we argue for case‐specific neuro *habilitatio*n; a process aimed at helping individuals born with, or incurring, pathologies that prevent them from functioning within‐range of societal norms and expectations.

To be clear, we do not espouse or suggest a reductionist view of morality—there is not one, isolated moral center of the brain, nor is there one consensual view of morality. In the next section we review empirical findings in support of our proposal for an *affective* moral focus, with proposed moral habilitation centered on stimulating and eliciting affective responses more consistent with those exhibited by the general population—affective responses that can be self‐reported (e.g., feelings of guilt or distress), physiologically detected (e.g., galvanic skin response), neurologically observed (e.g., fMRI studies), and qualitatively assessed (e.g., pro‐social, affiliative behaviors, interpersonal accountability).

Empirical findings (discussed in Diagnostic criteria for conduct disorder in Section 4) of reduced amygdala and ventromedial prefrontal cortex responsiveness to distress cues,^39^ the brain regions normally associated with affective response when witnessing others in pain,^40^ are consistent with our own clinical observations from decades of counseling at‐risk youth—that CD and ASPD individuals are lacking innate moral intuitions essential for conscience development. We would assert that, just as attempts to counsel someone who is paralyzed by debilitating anxiety is ineffective until pharmacologic intervention is introduced in conjunction with talk‐therapy, any continued attempts to counsel adolescents lacking prosocial moral intuitions about the importance of perspective‐taking, self‐reflection, and/or self‐regulation is an exercise in futility (for adolescent, and counselor alike) until these moral intuitions can be biochemically elicited or primed.

### VI.2 B. In support of an *affective* moral focus

Considerable work has highlighted the importance of empathic emotional responses in moral development,^41^ with a view that healthy individuals are predisposed to find the distress of others aversive. Advances in neuroscientific research are helping scientists and medical practitioners understand, observe, and potentially manipulate the biological processes involved in moral emotion and cognition. Functional magnetic resonance imaging (fMRI) scans of individuals with psychopathy show reduced autonomic responses when viewing images/videos of people being hurt or exhibiting emotional distress, and a reduced ability to differentiate sad and fearful expressions from other emotional states.^42^

Several studies using single photon emission computed tomography (SPECT) have found significant correlations between reduced prefrontal blood flow and increased antisocial, aggressive behaviors. Amplified MRI studies show significantly reduced prefrontal gray matter in antisocial and psychopathic individuals. Positron emission tomography (PET) studies show reduced glucose metabolism in the orbitofrontal and medial prefrontal cortices of impulsive patients and aggressive children.^43^ Scientists have discovered that the brain’s anterior cingulate cortex serves a connective function between the “emotional” limbic system and the “cognitive” prefrontal cortex, an “important role in integration of neuronal circuitry for affect regulation … identified as a distinctive region in understanding psychopathology” (p. 121).^44^

A number of researchers posit that emotions and even gut reactions play an essential role in constituting many moral judgments; if we “feel unease when contemplating an action, we judge it wrong, whereas a positively valenced feeling causes us to judge it right or at least permissible” (p. 115).^45^ Studies demonstrate that unconscious disgust responses can influence whether an act is perceived as morally wrong.^46^ Our experience of somatic states “orients us toward relevant stimuli and shapes our decisions” (p. 115).^47^

Understanding the biological basis of moral intuition and behavior helps shed light on why standard therapeutic approaches (e.g., cognitive behavioral therapy) remain ineffective treatments for these populations. These therapeutic strategies typically focus on correcting “faulty” cognitive reasoning processes related to moral judgments, but it has been demonstrated that psychopaths “show excellent (not poor) moral reasoning ability when discussing hypothetical situations” (p. 209).^48^ The missing factor is the *feeling* of what is moral in antisocial individuals, not the *knowing* of what is moral.^49^

As children grow and mature, so do their cognitive moral reasoning abilities; moral behaviors are understood to emerge from a dynamic of moral reasoning and moral emotions that “work in synergy and reciprocally influence each other” (p. 6).^50^ Conflicting moral decisions do not entail a conflict between emotion and cognition, but rather invoke both via “cortico‐limbic assemblies encoding distinct motivationally salient goals…to overcome a motivationally laden choice” (p. 164).^51^ It has been demonstrated that adolescents who do not systematically learn, form, and internalize moral standards are more “susceptible to external risk factors for deviance” (p. 5).^52^ Studies demonstrate that a youth’s initial onset of delinquency is linked with “moral insensitivity and internal pleasure” (p. 5)^53^ derived from risky behaviors, and continuity of delinquency further impacts moral development.

Individuals who can regulate their emotions are “more likely to experience empathy, and also to act in morally desirable ways with others” (p. 11).^54^ Chronic incapacity to suppress negative emotions is a key factor leading to aggressive and violent behaviors.^55^ There is an important distinction to be made between reactive and instrumental aggression; reactive aggression occurs as an explosive response to perceived threat or frustration and is not goal‐directed, whereas proactive or instrumental aggression is used to achieve a goal or elicit a reaction. While reactive aggression is common in most personality disorders, predatory aggression is more common in ASPD.^56^ Attributes of poor executive function and mood volatility do not lend themselves to successful predation. A predator exhibits patience and careful planning to disguise his or her intentions, chooses a vulnerable target, and strategizes the attack; calculated behaviors all driven by the expectation of gain or accomplishing a set goal.^57^ Distinct pathways for proactive and reactive aggression have been revealed, suggesting a stronger biological basis for proactive aggression than for reactive aggression, wherein environmental influences play a bigger role.^58^

### VI.3 C. Interventions

#### VI.3.1 Pharmacologic

A number of pharmaceuticals already being used on a regular basis affect human cognition and emotion. There have been trials of anticonvulsants, such as oxcarbazepine, as a means for treating aggressive outbursts in incarcerated populations.^59^ Additionally, for impulsive aggression, treatment with selective serotonin reuptake inhibitors has been found to increase glucose metabolism in the orbitofrontal cortex, improving regulatory functioning in regions that have been identified as deficient in criminal populations.^60^ Selective serotonin reuptake inhibitors (SSRIs) prescribed for the treatment of depression and anxiety, “seem to make subjects more cooperative and less critical of others” (p. 117)^61^ and also seem “to increase social affiliative behavior” (p. 117).^62^ Additional studies suggest that “potentiating serotonin increases aversion to directly causing harm to others” (p. 117).^63^

Atomoxetine and methylphenidate, both of which are indicated for attention deficit hyperactivity disorder (ADHD), are prescribed widely. Improving impulse control in ADHD “has significant effects on moral behavior…reducing risk of harm to others” (p. 112).^64^ The effects of other medications prescribed for various disorders, in mitigating symptoms of those disorders, potentially improve the moral behavioral responses of those people. For example, “anti‐craving, substitute and deterrent medications taken by those with addictions…the antilibidinal medicines administered to sex offenders” (p. 113)^65^ can also have morally significant effects.

Propranolol, a β‐blocker widely prescribed for the treatment of hypertension, has also been used, off‐label, to decrease performance anxiety and it has been investigated as a treatment or prophylactic for posttraumatic stress disorder (PTSD). PTSD, according to one widely accepted theory, arises from the over consolidation of traumatic memories.^66^ Propranolol acts to block the transmission of a neural signal by “blocking adrenergic receptors in the amygdala, a limbic brain region strongly linked with … emotion processing” (p. 5).^67^

While the focus of this paper is on “primary” psychopathy (instrumental aggression/CU subtype) with a strong genetic/biological underpinning, “secondary” psychopathy (reactive aggression) has a greater environmental influence.^68^ Early childhood stress and trauma, with inconsistent and/or harsh parenting styles, and maladjusted attachment can exacerbate and contribute to the antisocial behavioral responses in these populations. Many of these adolescents and young adults have concurrent diagnoses of PTSD; perhaps traumatic memory consolidation would prove beneficial in ameliorating dysfunctional interpersonal interactions (e.g., aggression towards people/places/events that elicit painful memories).

Oxytocin is produced naturally in the brain’s hypothalamus and released into both the brain and bloodstream. This hormone and neurotransmitter is best known for its somatic effects, facilitating birth and breastfeeding in humans and other mammals, but it may also influence morally significant behavior. For example, in (nonhuman) mammals, oxytocin seems to mediate pair bonding, maternal care, and other prosocial behaviors,^69^ and studies suggest that it plays a role in mediating trust, cooperation, empathy, and generosity in humans. Similarly, glucocorticoids, widely used to treat asthma and other disorders of inflammation, “are thought to modulate both the release and activity of oxytocin” (p. 118).^70^ ASPD individuals have deficits in recognizing fearful and happy facial expressions in others. Experiments of oxytocin administration in young adults with ASPD have helped them to recognize fearful and happy expressions, thus diminishing aggressive reactions and potentially increasing social reward responsiveness.^71^ In animal studies, oxytocin administration has served to normalize emotional contagion and decrease aggression, thereby ameliorating the phenotype of mice “characterised by abnormal aggression and excess callousness” (p. 251).^72^

The hypothesis of oxytocin increasing prosocial behaviors has been challenged by studies associating oxytocin with maladaptive behaviors. For example, one study demonstrated oxytocin administration heightened envy during a gambling game.^73^ Another study revealed increased plasma oxytocin levels in women experiencing emotional distress when encountering relationship threats to monogamous pair bonds.^74^ There has also been a concern raised that oxytocin’s increased trust effects may be sensitive to in‐group membership; e.g., in one study, participants administered intranasal oxytocin who viewed trolley dilemma scenarios were “significantly more likely to sacrifice a different race individual to save a group of race unspecified others than they were to sacrifice a same race individual” (p. 120),^75^ an in‐group bias not demonstrated by participants in the placebo group.

Another hormone and neurotransmitter recognized for its prosocial behavioral effects is the neuropeptide arginine vasopressin (AVP).^76^ AVP plays a key role in pair bond formation, parental care, and social approach across animal species. Empathic concern is the primary motivator of prosocial behavior and “multiple studies have shown that AVP increases human prosocial behavior” (p. 254).^77^ A randomized, double‐blind study tested the effects of intranasal administration of vasopressin compared to placebo on empathic responses, demonstrating that in “participants with higher levels of primary psychopathy vasopressin increased personal distress and empathic concern compared to placebo” (p. 58).^78^ Sex‐dependent evidence from animal and human studies demonstrates that while oxytocin is more influential in female prosociality, arginine vasopressin may play a greater role in stimulating male prosociality.^79^

Based on these studies, we would suggest that administration of oxytocin in women, and arginine vasopressin in men, can function as a prosocial “primer” wherein positive, affiliative behaviors can be elicited; the same prosocial default from which emotionally healthy individuals generally respond. Although some have argued that increased trust of others does not always result in good outcomes (e.g., increased trust can result in exploitation), adolescents and young adults exhibiting CD and/or ASPD seem, across the board, to be more distrusting and manipulative than others in their peer group, so it is unlikely that increasing trust levels in this population would become as problematic as increasing trust levels in a population already disposed to be trustful.

#### VI.3.2 Optogenetics

Optogenetics is an exciting neuromodulation technology that can manipulate biological processes via genetic modification of neurons to express light activated proteins (opsins), offering cell‐specific neural control with millisecond precision.^80^ Using this technique, recent studies have shown that specific neurons in the ventromedial hypothalamus (VMH) of mice, the region of the brain associated with satiety, can be manipulated in the regulation of male attack behaviors.^81^ Through optogenetic inhibition of the VMH, attack responses were suppressed; researchers succeeded in manipulating dramatic behavioral changes in mice “from the highly prosocial to the extremely antisocial” (p. 61).^82^ In another study, optogenetic stimulation of light‐sensitive protein located in the basolateral amygdala of mice reduced anxiety‐like behaviors and elicited an antidepressant‐like response.^83^ Unlike electroconvulsive therapy (ECT), administered in severe cases of pharmacoresistant depression, which indiscriminately stimulates large parts of the brain, optogenetics offers a much more targeted stimulation approach.^84^

#### VI.3.3 Other devices

In addition to hormonal and optogenetic modulation strategies, there may also be uses of brain stimulation devices to reduce impulsive or addictive behaviors and improve self‐control, thus “making associated ‘immoral behavior’ less likely” (p. 167).^85^ Research has shown that disruptive stimulation of the right prefrontal cortex or the temporoparietal junction can affect moral judgments relating to fairness and harm; though the “circumstances of these … investigations have been thus far largely contrived, such that the real‐world implications of the findings are not yet apparent” (p. 167).^86^ Repetitive transcranial magnetic stimulation (rTMS) studies use low frequency rTMS to create “a ‘functional lesion’ to test hypotheses regarding whether specific brain regions are necessary for specific moral judgments” (p. 2).^87^ Transcranial direct current stimulation (tDCS) studies examine whether increasing (anodal) or decreasing (cathodal) cortical excitability in a particular brain region will alter moral behavior. Anodal stimulation of the right dorsolateral prefrontal cortex (DLPFC) “increased decisions to trust and cooperate, indicating a general enhancement of prosocial behavior … (and) the willingness to intervene to help others in simulated situations” (p. 6).^88^

## VII ETHICAL CONSIDERATIONS

The first pharmacotherapeutic trials of childhood psychological disorders took place in the 1930/40s, when stimulants were administered to institutionalized hyperactive children.^89^ But, after WWII, and public exposure of the horrific Nazi war crimes of medical experimentation on psychiatric patients, sick children, and concentration camp victims, the Nuremberg Code was enacted, requiring stringent research protection protocols for any research conducted on human subjects, especially children and vulnerable populations. Due to these important safety measures, it is difficult to conduct the scope and breadth of research on children as can be done with adults. The most frequently prescribed substance classes to children and adolescents are stimulants, antidepressants, and second‐generation antipsychotics (SGAs), many of which are used off‐label. Antipsychotics are increasingly prescribed in the treatment of behavioral issues manifested by children.^90^

Ethical justifications supporting public health policy initiatives differ from those applied in individual patient determinations, due to how theories are interpreted. For example, in Western medicine, application of Beauchamp and Childress’ (2013)^91^ four principles of bioethics—autonomy, nonmaleficence, beneficence, and justice—will be assessed per a patient’s treatment needs, with emphasis on patient autonomy. However, the same four principles applied in the justification of what ought to be done to foster community safety and well‐being will emphasize justice concerns (e.g., equitable access to care), with nonmaleficence and beneficence determinations assessed in context of the public‐at‐large. In this section, we review public health‐focused ethical frameworks in support of compulsory moral neurohabilitation, while addressing patient‐specific ethical concerns as well.

Bioethicist Daniel Callahan (2003)^92^ applies the analogy of an ecosystem in espousing a communitarianism paradigm for public health ethics. When a new plant species is introduced into an ecosystem, the central question, according to Callahan, is whether it will live in harmony with, improve upon, or at the very least, bring no harm to the other inhabitants of the ecosystem.^93^ Communitarianism is premised on the fact that humans are social beings, embedded in communal institutions and practices. As such, Callahan cautions against overreliance on rationality and philosophical reasoning as drivers of human behavior, citing the roles emotion, moral imagination (e.g., in the form of empathy), and social reciprocity play in decision‐making.

Psychologist Carol Gilligan espouses a care ethics perspective, framing autonomy in “relational” terms, wherein individuals are inextricably linked to others in society (e.g., family members, significant others, peers, caretakers, etc.). When viewed through a relational autonomy perspective, it can be argued that notions of “care and coercion are not adversaries, but are … linked when people have to rely on each other for support and safety” (p. 83).^94^ A care ethics approach would justify case‐specific compulsory treatment when an individual’s dysfunctional behavior negatively impacts other family members, significant others, or community members within the individual’s sphere of interaction and influence.

In utilitarian, outcome‐based justifications for public health initiatives, policies are proposed, monitored, and empirically measured with a view towards maximization of health promotion and safety across the population. As such, threshold determinations are made as to which health measures can remain voluntary ones, and which cannot—as in the compulsory quarantining of individuals with infectious disease to prevent widespread community transmission, or the passage of involuntary outpatient commitment (OPC) legislation, wherein a civil court order mandates treatment compliance for seriously mentally ill individuals living in the community, either post‐hospitalization or as an alternative to hospitalization.^95^

Opponents to mandated mental health treatment programs cite liberty and autonomy‐infringement concerns, to which we would respond: (a) mandated outpatient treatment is a less restrictive alternative to inpatient hospitalization or incarceration, and (b) such individuals are in actuality lacking autonomy when devoid of reality awareness (e.g., psychosis), behavioral insight, or accountability, all of which are essential for decision‐making. Indeed, when antisocial behaviors result in school suspension/expulsion, work discharge, interpersonal strife, legal trouble and even incarceration, it can be argued that moral neurohabilitation would potentially increase individual autonomy and options for improved educational, employment, and relationship opportunities.

Justice arguments in support of mandated neurohabilitation include increased societal safety, and decreased reliance on emergency medical and law enforcement personnel, freeing up these valuable resources to others who may need them. To ensure that prejudice and bias do not impact case‐specific determinations, criteria for compulsory moral habilitation would have to be clearly defined and consistently implemented, determinations would be made by more than one licensed mental health professional, and there would be ongoing monitoring and accountability. Additionally, treatment access and cost‐coverage must be assured through federal and state funded programs.

Would moral neurohabilitation change the person’s identity? This is a challenging question, as interpretations of personhood and identity vary based on philosophical framework, psychological construct, socio‐cultural lens, or legal definition applied. Personhood or identity can be framed philosophically (e.g., ability to reason or reflect), psychologically (e.g., self‐identity narrative, personality traits), morally (e.g., value systems, behaviors), relationally (e.g., cultural/social embeddedness), biologically (e.g., system function, sentience, physiology, genetic composition), and legally (e.g., entity, rights‐bearer, contract participant). Conflicting opinions abound as to whether one becomes “a different person” following certain medical interventions, events, or experiences, such as neurological procedures (e.g., deep‐brain stimulation);^96^ neurodegenerative disease (e.g., Alzheimer’s);^97^ life experiences (e.g., emotional trauma);^98^ addictive behaviors (e.g., substance abuse);^99^ or medication administration (e.g., attention deficit hyperactivity disorder/ADHD).^100^

A study exploring the change‐in‐identity question conducted by Strohminger and Nichols (2014)^101^ revealed that when study participants read vignettes of people who manifested behavior changes due to four conditions—agnosia (lost ability to recognize objects), apathy (lost desire), amnesia (lost memory), or morality (lost moral conscience)—with participants asked to comment on which individuals exhibited the greatest identity change, there was strong agreement that vignettes describing people who lost their “moral conscience” were “considered to be more of a different person than in any other condition” (p. 124).^102^ Other studies have been conducted^103^ with similar results, wherein moral traits are ascribed as essential to personal identity. Based on these findings, it might be deduced that someone gaining “moral conscience” following treatment would also be viewed as exhibiting significant identity change. However, self‐ and other‐views of identity change, and accompanying assessments of whether such changes are good or bad, welcome or unwelcome, will depend upon whose lens, and in what context, these evaluations are being made. Lacking capacity for self‐insight, the ASPD individual’s determinations of better or worse will have no baseline points of reference from which to make comparisons, making such assessments less reliable than those of family members, peers, and/or and others in the community with whom the individual interacts.

The informed‐consent guidelines of the American Academy of Pediatrics (2016)^104^ recommend practitioner efforts to obtain child and adolescent assent, in addition to parental/guardian consent, for medical treatment or research participation. This not only helps ensure treatment compliance, but validates an adolescent’s autonomy for decision‐making.^105^ In recognition of increased adolescent decision‐making capacity, “Mature Minor” legislation has been enacted in many U.S. states, granting permission for adolescents to seek out reproductive health services, substance abuse and mental health treatments without parental consent.^106^ In some circumstances, the courts have upheld adolescents’ rights to refuse invasive or chronic medical treatments/interventions dependent on case‐based determinations of cognitive/emotional maturity.^107^ But placing high dependence on adolescent decision‐making poses problems when there is little incentive for the adolescent to seek treatment on her own (e.g., substance abuse) or when there is little to no insight, or personal accountability, of maladaptive behaviors impairing daily functioning, socialization, and emotional growth.

We contend that in applying risk‐benefit assessments weighting potential harms of autonomy‐infringement against those of community harms, some factors *can* be controlled, while others *cannot*—specifically, society *can* institute and monitor stringent procedural safeguards against individual rights‐infringement, whereas society *cannot*, post‐facto, restore the lives lost or fully ameliorate the trauma incurred by family members and friends of victims. Mental health practitioner “duty to warn”^108^ can only legally be invoked when a patient articulates a specific plan of inflicting imminent harm upon a specified individual. This legal mandate is insufficient, leaving desperate family members and trained clinicians frustrated and disempowered; individuals with these diagnoses do not seek out (or continue) treatment, do not articulate to others their specific plans to harm, and do not experience normative psychophysiological warning systems in the form of moral distress, guilt, or interpersonal accountability that would serve to thwart these dangerous impulses.

## VIII DUAL ROLE CHALLENGES

Mental health practitioners, especially those working in a forensics capacity (i.e., psychiatrists), are tasked with the dialectical tension of maintaining a therapeutic alliance with patients, while simultaneously serving as court consultants rendering determinations of culpability in criminal proceedings or dangerousness to society leading to involuntary holds. Which of these “dual role” or “dual loyalty” dilemmas^109^ responsibilities takes priority will depend on various factors, ranging from the views of individual practitioners, to those of employer/institutional expectations, to legal/safety considerations, etc. In a recent qualitative study, wherein forensic psychiatrists were asked whether “stimulating moral development or moral growth is or should be part of forensic psychiatric treatment” (p. 75),^110^ many respondents answered that while morality and conscience development are implicit aspects of treatment, they are not overt. Some expressed fears of misconception about the scope or expected outcomes of their services, while others cited concerns about imposing personal beliefs or value judgments. But we would remind practitioners that there are times when beneficent care requires non‐neutrality. This holds true both in medical and mental health care contexts. Not only is it unrealistic for practitioners to maintain a wholly non‐judgmental stance towards the psychopathic patients they treat, these pronouncements pose different kinds of challenges; specifically, implicit rather than overt treatment objectives can undermine the physician‐patient relationship, maintaining a professional balancing act of “dual loyalty” contributes to practitioner feelings of emotional distress and burnout, and, by not openly and explicitly acknowledging a treatment goal of morality/conscience development, there are missed opportunities for dedicated research/funding towards this area of treatment potential.

## IX CONCLUSION

When behaviors or actions jeopardize the health and safety of society‐at‐large, public health laws and procedures can override individual autonomy for the greater good. In any treatment provision (compulsory or not), the benefits of medication and/or technology use must always outweigh the harms. There is much more research needed regarding uses and effectiveness of any of the moral neurohabilitative options reviewed here. The most immediately translatable option would be SSRIs or other medications already utilized, but with research dedicated to testing for increased moral, prosocial effects. We also recommend research on the administration of oxytocin, or arginine vasopressin, in conjunction with counseling, to determine whether affective “priming” helps CD and ASPD adolescents/young adults to exhibit affiliative, intuitively driven moral behaviors. A holistic approach to any medical treatment is prudent; experimental clinical neuromodulation protocols should always be accompanied by counseling, case monitoring, and patient feedback. Perhaps one day in the not too distant future, adolescents and adults diagnosed with CD and ASPD may finally be helped to care about others, enjoying the rewards that come with healthy interpersonal interactions, empathic concern, and social affiliation. And the people with whom they interact will no longer have to live in fear.

## CONFLICT OF INTEREST

The authors declare no conflict of interest.

## Footnotes

^1^
Weiner, D.B. (2008). The Madman in the Light of Reason, Chapter 6 (pp. 255‐281). In Wallace, E.R. & Gach, J. (Eds.) *History of Psychiatry and Medical Psychology*. New York, New York: Springer Science and Business Media.

^2^
Scanlon, E. (2007). Willowbrook State School. In A. Bursztyn (Ed.), *The Praeger handbook of special education* (pp. 10–12). Connecticut: Greenwood Publishing Group.

^3^
Van de Vandervoort, J. W., & Hamlin, J. K. (2016). Evidence for intuitive morality: Preverbal infants make sociomoral evaluations. Child Development Perspectives, 10(3), 143–148. https://doi.org/10.1111/cdep.12175

^4^
Hamlin, J. K. (2015). The infantile origins of our moral brains. In J. Decety & T. Wheatley (Eds.), The moral brain—Multidisciplinary perspectives (pp. 105–122). Cambridge, MA: MIT Press; Hamlin, J. K., Wynn, K., & Bloom, P. (2010). Three‐month‐olds show a negativity bias in their social evaluations. Developmental Science, 13, 923–929. https://pnas.org/cgi/doi/10.1073/pnas.111030610810.1111/j.1467-7687.2010.00951.xPMC296603020977563

^5^
Decety, J., & Cowell, J. M. (2014). The complex relationship between morality and empathy. Trends in Cognitive Sciences, 18, 337–339. https://doi:10.1016/j.tics.2014.04.0810.1016/j.tics.2014.04.00824972506

^6^
Decety, J., & Cowell, J. M. (2018). Interpersonal harm aversion as a necessary foundation for morality: A developmental neuroscience perspective. Development and Psychopathology, 30 (1), 153–164. https://10.1017/S0954579417000530; Hamlin et al., op. cit. note 4.10.1017/S095457941700053028420449

^7^
Decety & Cowell, op. cit. note 6.

^8^
Van de Vandervoort & Hamlin, op. cit. note 3.

^9^
Decety & Cowell, op. cit. note 6.

^10^
Decety, J., Michalska, K. J., & Kinzler, J. D. (2012). The contribution of emotion and cognition to moral sensitivity: A neurodevelopmental study. *Cerebral Cortex*, *22*, 209–220. 10.1093/cercor/bhr11 21616985

^11^
Ibid.

^12^
Decety & Cowell, op. cit. note 6.

^13^
Balia, C., Carucci, S., Coghill, D., & Zuddas, A. (2016). The pharmacological treatment of aggression in children and adolescents with conduct disorder. Do callous‐unemotional traits modulate the efficacy of medication? *Neuroscience and Biobehavioral Reviews*, *91*, 218–238. 10.1016/j.neubiorev.2017.01.024 28137460

^14^
American Psychiatric Association. (2013). *Diagnostic and statistical manual of mental disorders (DSM‐5)* (5th ed.). Washington D.C.: American Psychiatric Association.

^15^
Balia et al., op. cit. note 13.

^16^
Ibid; Blair, R. J. R. (2013). The neurobiology of psychopathic traits in youths. *Nature Reviews Neuroscience, 14, 786‐789*. 10.1038/nrn3577 PMC441850724105343

^17^
Hawes, D., Price, M., & Dadds, M. (2014). Callous unemotional traits and the treatment of conduct problems in childhood and adolescence: A comprehensive review. *Clinical Child and Family Psychology Review, 17*(3), 248–267. 10.1007/s10567-014-0167-1 24748077

^18^
Ibid.

^19^
Balia et al., op. cit. note 13; Blair, op. cit. note 16.

^20^
Whittle, S., Liu, K., Bastin, C., Harrison, B. J., & Davey, C. G. (2016). Neurodevelopmental correlates of proneness to guilt and shame in adolescence and early adulthood. *Developmental Cognitive Neuroscience, 19*, 51–57. 10.1016/j.dcn.2016.02.001 PMC699009426895352

^21^
Ibid.

^22^
Ibid.

^23^
Ibid.

^24^
Salisbury, T. (2014). The relationship between oppositional defiant disorder, conduct disorder, antisocial personality disorder and psychopathy: A proposed trajectory. *Western Undergraduate Psychology Journal, 1*(1), 1‐9. https://ir.lib.uwo.ca/wupj/vol1/iss1/2

^25^
Ibid.

^26^
Glenn, A. L., Johnson, A. K., & Raine, A. (2013). Antisocial personality disorder: A current review. *Current Psychiatry Reports, 15*(12). 10.1007/s11920-013-0427-7 24249521

^27^
Ibid.

^28^
Ibid.

^29^
Skeem, J., Johansson, P., Andershed, H., Kerr, M., & Louden, J. E. (2007). Two subtypes of psychopathic violent offenders that parallel primary and secondary variants. *Journal of Abnormal Psychology, 116*(2), 395–409. 10.1037/0021-843X.116.2.395 17516770

^30^
Ibid.

^31^
Brazil, I. A., van Dongen, J. D. M., Maes, J. H. R., Mars, R. B., & Baskin‐Sommers, A. R. (2016). Classification and treatment of antisocial individuals: From behavior to biocognition. *Neuroscience and Biobehavioral Reviews, 91*, 259–277. 10.1016/j.neurobiorev.2016.10.010 27760372

^32^
Simone, S., & Fulero, S. M. (2005). Tarasoff and the duty to protect. *Journal of Aggression, Maltreatment & Trauma, 11*(1–2), 145–168. 10.1300/JI46v11n01_12

^33^
Howells, K., Krishnan, G., & Daffern, M. (2007). Challenges in the treatment of dangerous and severe personality disorder. *Advances in Psychiatric Treatment, 13*(5), 325–332. 10.1192/apt.bp.106.002857

^34^
Ibid.

^35^
Ibid.

^36^
Wiseman, H. (2016). *The myth of the moral brain: The limits of moral enhancement* (Kindle edition). Boston, MA: MIT Press.

^37^
Ibid.

^38^
Persson, I., & Savulescu, J. (2008). The perils of cognitive enhancement and the urgent imperative to enhance the moral character of humanity. *Journal of Applied Philosophy, 25*, 162–177. 10.1111/j.1468-5930.2008.00410.x

^39^
Balia et al., op. cit. note 13; Blair, op. cit. note 16.

^40^
Decety & Cowell, op. cit. note 6; Marsh, A. A., Finger, E. C., Fowler, K. A., Adalio, C. J., Jurkowitz, I. N., Schechter, J. C., … Blair, R. J. R. (2013). Empathic responsiveness in amygdala and anterior cingulate cortex in youths with psychopathic traits. *Journal of Child Psychology and Psychiatry, 54*, 900–910. 10.1111/jcpp.12063 PMC371683523488588

^41^
Blair, R. J. R. (2007). The amygdala and ventromedial prefrontal cortex in morality and psychopathy. *Trends in Cognitive Sciences*, *11*(9), 387–392. 10.1016/j.tics.2007.07.003 17707682

^42^
Ibid.

^43^
Raine, A., & Yang, Y. (2006). Neural foundations to moral reasoning and antisocial behavior. *Social Cognitive and Affective Neuroscience, 1*(3), 203–213. 10.1093/scan/nsl033 PMC255541418985107

^44^
Stevens, F. L., Hurley, R. A., & Taber, K. H. (2011). Anterior cingulate cortex: Unique role in cognition and emotion. *The Journal of Neuropsychiatry and Clinical Neurosciences, 23*(2), 121–125. 10.1176/jnp.23.2.jnp121 21677237

^45^
Levy, N., Douglas, T., Kahane, G., Terbeck, S., Cowen, P. J., Hewstone, M., & Savulescu, J. (2014). Are you morally modified?: The moral effects of widely used pharmaceuticals. *Philosophy, Psychiatry, & Psychology*, *21*(2), 111–125. 10.1353/pp.2014.0023 PMC439897925892904

^46^
Ibid; .Haidt, J. & Joseph, C. (2007). The moral mind: How five sets of innate intuitions guide the development of many culture‐specific virtues, and perhaps even modules. In P. Carruthers, S. Laurence, & S. Stich (Eds.), *The innate mind*, Vol. 3. Foundations and the future. Oxford University Press. 10.1093/acprof:oso/9780195332834.001.0001

^47^
Levy et al., op. cit. note 45.

^48^
Raine & Yang, op. cit. note 43.

^49^
Ibid.

^50^
Jotterand, F. (2011). “Virtue Engineering” and moral agency: Will post‐humans still need the virtues? *AJOB Neuroscience, 2*(4), 3–9. 10.1080/21507740.2011.611124

^51^
Moll, J., De Oliveira‐Souza, R., & Zahn, R. (2008). The neural basis of moral cognition: Sentiments, concepts, and values. *Annals of the New York Academy of Sciences, 1124*, 161–180. 10.1196/annals.1440.005 18400930

^52^
Du, Y. (2019). Developing an integrated biosocial theory to understand juvenile delinquency: From the social, cognitive, affective, and moral (SCAM) perspectives. *International Journal of Contemporary Pediatrics, 6*(2), 1‐7. 10.18203/2349-3291.ijcp20190751

^53^
Ibid.

^54^
Decety, J., & Moriguchi, Y. (2007). The empathic brain and its dysfunction in psychiatric populations: Implications for intervention across different clinical conditions. *BioPsychoSocial Medicine, 1*(1), 1‐22. 10.1186/1751-0759-1-2 PMC220603618021398

^55^
Ibid.

^56^
Svrakic, D., Divac‐Jovanovic, M., & Azhar, N. (2019). An integrative model and dynamic nosology of personality disorder: Part 2: Symptom‐based pharmacotherapy. *Psychiatria Danubina, 31*(1), 2–17. 10.24869/psyd.2019.2 30948684

^57^
Ibid.

^58^
Fragkaki, I., Cima, M., Verhagen, M., Maciejewski, D. F., Boks, M. P., van Lier, P. A. C., … Meeus, W. H. J. (2019). Oxytocin receptor gene (OXTR) and deviant peer affiliation: A gene‐environment interaction in adolescent antisocial behavior. *Journal of Youth and Adolescence, 48*(1), 86–101. 10.1007/s10964-018-0939-x 30315439

^59^
Brazil et al., op. cit. note 31.

^60^
Glenn, A., & Raine, A. (2009). Psychopathy and instrumental aggression: Evolutionary, neurobiological, and evolutionary perspectives. *International Journal of Law and Psychiatry, 32*(4), 253‐258. 10.1016/j.ijlp.2009.04.002 19409615

^61^
Levy et al., op. cit. note 45.

^62^
Ibid.

^63^
Ibid.

^64^
Ibid.

^65^
Ibid.

^66^
Ibid.

^67^
Ibid.

^68^
Glenn & Raine, op. cit. note 60.

^69^
Levy et al. op. cit. note 45.

^70^
Ibid.

^71^
Timmerman, M., Jeung, H., Schmitt, R., Boll, S., Freitag, C. M., Bertsch, K., & Herpertz, S. C. (2017). Oxytocin improves facial emotion recognition in young adults with antisocial personality disorder. *Psychoneuroendrocrinology, 85, 158‐164.* 10.1016/j.psyneuen.2017.07.483 28865940

^72^
Zoratto, F., Sbriccoli, M., Martinelli, A., Glennon, J. C., Macri, S., & Laviola, G. (2018). Intranasal oxytocin administration promotes emotional contagion and reduces aggression in a mouse model of callousness. *Neuropharmacology, 143*(September), 250–267. 10.1016/j.neuropharm.2018.09.010 30213592

^73^
Shamay‐Tsoory, S. G., Fischer, M., Dvash, J., Harari, H., Perach‐Bloom, N., & Levkovitz, Y. (2009). Intranasal administration of oxytocin increases envy and Schadenfreude (gloating). *Biological Psychiatry*, *66*(9), 864–870. 10.1016/j.biopsych.2009.06.009 19640508

^74^
Tabak, B. A., Meyer, M. L., Castle, E., Dutcher, J. M., Irwin, M. R., Han, J. H., & Eisenberger, N. I. (2015). Vasopressin, but not oxytocin, increases empathic concern among individuals who received higher levels of paternal warmth: A randomized controlled trial. *Psychoneuroendocrinology, 51*, 253–261. 10.1016/j.psyneuen.2014.10.006; Taylor, S. E., Saphire‐Bernstein, S., & Seeman, T. E. (2010). Are plasma oxytocin in women and plasma vasopressin in men biomarkers of distressed pair‐bond relationships? *Psychological Science, 21*(1), 3–7. 10.1016/j.psyneuen.2014.10.006

^75^
Levy et al. op. cit. note 45.

^76^
Tabak et al., op. cit. note 74.

^77^
Ibid.

^78^
Han, J. H., Tabak, B. A., Meyer, M. L., Castle, E., Dutcher, J. M., Irwin, M. R., … Eisenberger, N. (2016). PT710. Vasopressin increases empathic responding among those high in primary psychopathy. *International Journal of Neuropsychopharmacology, 19*(Suppl. 1), 58–59. 10.1093/ijnp/pyw044.710

^79^
Andreou, D., Comasco, E., Åslund, C., Nilsson, K. W., & Hodgins, S. (2018). Maltreatment, the oxytocin receptor gene, and conduct problems among male and female teenagers. *Frontiers in Human Neuroscience*, *12*(March), 1–9. 10.3389/fnhum.2018.00112 PMC587449529623035

^80^
Deng, C., Yuan, H., & Dai, J. (2018). Behavioral manipulation by optogenetics in the nonhuman primate. *Neuroscientist, 24*(5), 526–539. 10.1177/1073858417728459 28874078

^81^
Bartholow, B. D. (2018). The aggressive brain: Insights from neuroscience. *Current Opinion in Psychology, 19*, 60–64. 10.1016/j.copsyc.2017.04.002 29279225

^82^
Ibid.

^83^
Greenberg, A. (2018). Lighting a path to precision. Washington, D.C.: Niskanen Center. Retrieved from https://niskanencenter.org/blog/lighting‐a‐path‐to‐precision‐medicine

^84^
Ibid.

^85^
Johnson, L. S. M., Rommelfanger, K. S., Earp, B. D., Douglas, T., & Savulescu, J. (2018). *The Routledge handbook of moral neurohenhancement, United Kingdom: Routledge Taylor & Francis Group*

^86^
Ibid.

^87^
Darby, R. R., & Pascual‐Leone, A. (2017). Moral enhancement using non‐invasive brain stimulation. *Frontiers in Human Neuroscience, 11*(77), 1‐10. https://www.frontiersin.org/article/10.3389/fnhum.2017.00077 10.3389/fnhum.2017.00077PMC531998228275345

^88^
Ibid.

^89^
Koelch, M., Schnoor, K., & Fegert, J. M. (2008). Ethical issues in psychopharmacology of children and adolescents. *Current Opinion in Psychiatry, 21(6), 598‐605.* 10.1097/YCO.0b013e328314b776 18852568

^90^
Ibid.

^91^
Beauchamp, T. L., & Childress, J. F. (2013). *Principles of biomedical ethics* (7th ed.). New York, NY: Oxford University Press.

^92^
Callahan, D. (2003). Principlism and communitarianism. *Journal of Medical Ethics, 29*(5), 287–291. 10.1136/jme.29.5.287 PMC173378714519838

^93^
Ibid.

^94^
Adshead, G., & Davies, T. (2016). Wise restraints: Ethical issues in the coercion of forensic patients. In B. Vollm & N. Nedopil (Eds.), *The use of coercive measures in forensic psychiatric care: Legal, ethical, and practical challenges* (pp. 69–86). Switzerland: Springer International Publishing Group.

^95^
Swartz, M., & Swanson, J. (2019). Mandated community treatment in services for persons with mental illness. In Goldman, H.H., Frank, R.G., & Morrissey, J.P. (Eds) *The Palgrave handbook of mental health policy* (pp. 171–196). New York, New York: Palgrave Macmillan.

^96^
Goddard, E. (2017). Deep brain stimulation through the “lens of agency”: Clarifying threats to personal identity from neurological intervention. *Neuroethics, 10*(3), 325–335. 10.1007/s12152-016-9297-0

^97^
Naue, U., & Kroll, T. (2009). ‘The demented other’: Identity and difference in dementia. *Nursing Philosophy, 10*(1), 26–33. 10.1111/j.1466-769X.2008.00379.x 19154294

^98^
Muldoon, O. T., Haslam, S. A., Haslam, C., Cruwys, T., Kearns, M., & Jetten, J. (2019). The social psychology of responses to trauma: Social identity pathways associated with divergent traumatic responses. *European Review of Social Psychology, 30*(1), 311–348. 10.1080/10463283.2020.1711628

^99^
Earp, B. D., Skorburg, J. A., Everett, J. A. C., & Savulescu, J. (2019). Addiction, identity, morality. *AJOB Empirical Bioethics, 10*(2), 136–153. 10.1080/23294515.2019.1590480 PMC650690731012802

^100^
Jones, S., & Hesse, M. (2018). Adolescents with ADHD: Experiences of having an ADHD diagnosis and negotiations of self‐image and identity. *Journal of Attention Disorders, 22*(1), 92–102. 10.1177/1087054714522513 24550560

^101^
Strohminger, N., & Nichols, S. (2014). The essential moral self. *Cognition, 131*(1), 159–171. 10.1016/j.cognition.2013.12.005 24503450

^102^
Skorburg, J. A., & Sinnott‐Armstrong, W. (2020). Some ethics of deep brain stimulation. In Stein, D.J. & Singh, I. (Eds.) *Global Mental Health and Neuroethics*, 117–132. United Kingdom: Elsevier https://philarchive.org/archive/AUGSEOv1

^103^
Heiphetz, L., Strohminger, N., Gelman, S. A., & Young, L. L. (2018). Who am I? The role of moral belief in children’s and adult’s understanding of identity. *Journal of Experimental Social Psychology, 78*, 2010–2019. 10.1037/xge0000587

^104^
American Academy of Pediatrics. (2016). Informed consent in decision‐making in pediatric practice. https://pediatrics.aappublications.org/content/138/2/e20161484 10.1542/peds.2016-148527456510

^105^
Katz, A. L., & Webb, S. A. (2016). Informed consent in decision‐making in pediatric practice. *Pediatrics,* 138(2), e20161485. 10.1542/peds.2016-1485 27456510

^106^
Coleman, D. L., & Rosoff, P. M. (2013). The legal authority of mature minors to consent to general medical treatment. *Pediatrics, 131*(4), 786–793. 10.1542/peds.2012-2470 23530175

^107^
Penkower, J. A. (1996). The potential right of chronically ill adolescents to refuse life‐saving medical treatment‐‐fatal misuse of mature minor doctrine. *De Paul Law Review, 45*(4), 1165–1216. https://via.library.depaul.edu/law‐review/vol45/iss4/10 16977737

^108^
Felthous, A. R. (2006). Warning a potential victim of a person’s dangerousness: Clinician’s duty or victim’s right? *Journal of the American Academy of Psychiatry and the Law, 34*(3), 338–348. http://jaapl.org/content/jaapl/34/3/338.full.pdf 17032958

^109^
Specker, J., Focquaert, F., Sterckx, S., & Schermer, M. H. N. (2018). Forensic practitioners’ views on stimulating moral development and moral growth in forensic psychiatric care. *Neuroethics*, 13, 73‐85. 10.1057/s41292-017-0069-9

^110^
Ibid.

